# Assessing the Reliability and Validity of the Spanish Version of the Actual Scope of Nursing Practice Scale

**DOI:** 10.3390/healthcare11081170

**Published:** 2023-04-19

**Authors:** Amaia Saralegui-Gainza, Nelia Soto-Ruiz, Paula Escalada-Hernández, Cristina García-Vivar, Diego Rivera, Leticia San Martín-Rodríguez

**Affiliations:** Department of Health Sciences, Public University of Navarre (UPNA), 31008 Pamplona, Navarra, Spain; amaia.saralegui@gmail.com (A.S.-G.); paula.escalada@unavarra.es (P.E.-H.); cristina.garciavivar@unavarra.es (C.G.-V.); diegoriveraps@gmail.com (D.R.); leticia.sanmartin@unavarra.es (L.S.M.-R.)

**Keywords:** scope of practice, nursing management, psychometric properties, cross-cultural adaptation, validation

## Abstract

Nursing workforce shortage is one of the main challenges for healthcare organizations and it is important to determine if nurses are fulfilling their full scope of practice. There is a questionnaire that measures the activities carried out by nurses, but there is no version available for the Spanish context. The purpose of this study was to develop a cross-cultural adaptation of the “Actual Scope of Nursing Practice” questionnaire by D’Amour et al., and to assess the psychometric characteristics of the Spanish version. An exploratory sequential research design was used. The cross-cultural adaptation was performed using translation, back-translation, review, and pre-testing. Psychometric properties were assessed to determine its construct validity and internal consistency. Among the 501 eligible nurses from the three main hospitals in the region, the first 310 nurses to respond to an online questionnaire were included in our study. The response rate was 61.9%. They were invited via email and completed the survey using the SurveyMonkey platform. The Spanish version of the questionnaire was obtained. A final scale with twenty items and two factors was confirmed with an adequate fit, with the item scores demonstrating that all items were optimally related to their respective latent construct. The alpha coefficients for the Spanish ASCOP scale were robust and revealed good internal consistency. This study showed that the Spanish version of the scale, “Scope of Nursing Practice”, has a good degree of validity and reliability. This questionnaire can support nurse managers in realising nursing activities within their organisations and in promoting desirable work outcomes among nurses.

## 1. Introduction

One of the most important challenges for health care organisations is nursing workforce management. Today, the nursing workforce represents the highest human resource and involves more than 60% of the total volume of health professionals [1,2], therefore the correct management and optimal use of the nursing workforce is key to ensuring an efficient distribution for the health care system [3].

Nurses stop performing activities that add value to patient care and, on the other hand, tend to take on tasks that could be delegated to other professionals [2]. In fact, nurses spend more time performing indirect activities related to care coordination and quality of care while “less than one third work time is spent with patients” [4] (p. 4464).

To achieve greater efficiency of the nursing workforce, the tasks that particularly belong to nurses should be recognised and distinguished. According to D’Amour et al. (2012) [5], the range of activities carried out by nurses, also called “the scope of nursing practice”, implies “the range of functions and responsibilities carried out by nurses in relation to their skills acquired through their training and experience” [5] (p. 249).

It is essential to be able to quantify to what extent nurses’ functions are being carried out and to know what the factors or determinants that predict, facilitate, and/or hinder the effectiveness of nursing resources are. It is also true that the scope of nursing practice differs from one country to another, essentially due to their versatility and high flexibility. In addition, a nurse’s work can be structured based on different levels of training in the same country and even by the existence of different degrees between countries, where activities are not always well defined [6]. Therefore, having a tool to measure nurses’ activities and responsibilities in practice can help health care managers to plan, execute, and control all aspects of the management process.

Several tools that have been used to gauge the scope of nursing practice. The scale “Scope and Standards of Practice” [7] is one of the most widely used instruments in the American literature and is supported by the American Nursing Association. These standards serve to define nursing professional development and are also helpful in identifying a novice nurse’s path to becoming an expert nurse [8]. Other questionnaires have been identified that establish the nurses’ scope of practice from a nursing specialty perspective; for example, in cardiology nursing [2] and also the “Scope of School Nursing Practice Tool” for school nurses [9]. From a general perspective of the scope of nursing practice, other scales such as the “Implementation of scope of practice” [10] or “Scope-OAU” [11] have been used in studies in Israel and Australia, respectively. In the Canadian context, D’Amour et al. (2012) developed the “Actual Scope of Nursing Practice” (ASCOP) questionnaire to measure the scope of nursing practice, to assess the nature of the activities carried out by nurses and to determine the level of complexity of these activities [5]. Thus, the ASCOP provides a differential perspective in relation to other instruments.

The original ASCOP questionnaire was developed in English and French and consists of a twenty-six-item Likert-type scale questionnaire that is grouped into six dimensions related to: (1) the assessment and planning of care; (2) patient and family education; (3) communication and coordination of care; (4) integration and supervision of personnel; (5) quality and safety; and (6) updating and use of knowledge [5]. 

In addition to the dimensions, each activity is labelled with reference to a level of complexity (from level 1 or low complexity to level 3 or highly complex activities) [5]. Each item is answered on a six-point Likert frequency scale, which ranges from “never” (minimum score) to “always” (maximum score). The total score is obtained by adding the answers, considering 1 as the smallest scope of practice and 6 as wide as possible for the scope of practice. In relation to internal consistency identified by the original study, the raw coefficient alpha for the 26 items together was 0.89, with values for individual dimensions ranging from 0.61 to 0.70 for the six subscales. Otherwise, the principal component analysis (PCA) showed that the first principal factor explained 59% of the overall variation of the ASCOP tool, as well as variances between 40% and 62% [5].

In addition to the original ASCOP study developed in Canada, an Arabic version of the ASCOP (A-ASCOP) was formulated, offering an acceptable goodness-of-fit of a unidimensional model with all twenty five A-ASCOP items and the three levels of the item complexity model reported in the original instrument [12]. The Arabic version of the questionnaire included 25 items that were grouped into a unique dimension and was found to be valid and reliable, and presented high internal consistency with a raw coefficient alpha of 0.93 [13].

The original ASCOP questionnaire was designed in French [14] and was subsequently published and validated in English [5] and Arabic [13]. However, no instrument has been validated to establish the scope of nursing practice in the Spanish context. Thus, adequate assessment or articulation of the extent to which Spanish registered nurses apply the breadth of their scope of practice is not possible. Furthermore, we are unable to address the potential underutilisation of the nursing workforce.

Hence, given the importance of having a valid and reliable instrument to determine the scope of practice among nurses in hospitalization units, the aim of this study was to foster the cross-cultural adaptation and assess the psychometric characteristics of the Spanish version of the ASCOP scale by D’Amour et al. [5].

## 2. Methods

This methodological study was performed in two stages. First, the original instrument was translated and culturally adapted to the Spanish language. Second, the psychometric properties of the Spanish version of the ASCOP scale were assessed, by means of analysing their construct validity and determining the internal consistency of the statements. The study followed the STROBE checklist for observational studies.

### 2.1. Phase 1. Traslation and Cultural Adaptation of the Instrument

The initial stage involved translating and adapting the item statements from English into Spanish (spoken in Spain), using the process suggested by Mason [15]. The statements for the instrument were consequently translated and back-translated following the method proposed by Brislin [16]. The original English scale was used because the authors of the questionnaire reported identical psychometric characteristics in both French and English versions and because it is the one used for other adaptations to other languages and contexts. First, a Spanish-speaking person who was familiar with the health care terminology used completed an initial translation of the questionnaire into Spanish. An assessment of the translation was carried out with the objective of determining the equivalence of the meaning of the items on the two questionnaires. Four nurses with mastery of the original language of the questionnaire, who were Spanish-speaking and familiar with the terminology in the scientific literature in the field of health, participated in this assessment. Second, to ensure linguistic validation, another bilingual professional, independent from the first, English-speaking and specialised in the health sciences, performed the retro-translation from Spanish to English [17]. Then, a comparison was made between the original version of the questionnaire and the back translation, through a five-member panel of experts with experience in relevant areas of nursing management and familiar with the methods and terminology used, who discussed the differences found and reached agreement. Third, a pilot study was used to verify the applicability of the instrument and the quality of the translation. For this, five nurses with experience in the field of nursing management, as well as in the psychometric analysis of the instruments, were selected to perform the pre-test of understanding the items. They were asked to indicate which items that could be confusing and to add an alternative in cases where appropriate. No major changes were proposed. 

### 2.2. Phase 2. Analysis of the Psychometric Characteristics of the Instrument

In the second stage, an evaluation was performed on the questionnaire’s degree of validity and reliability by means of determining the construct validity and the internal consistency of the statements.

#### 2.2.1. Participants

The unit managers collaborated in sending the e-mail and nurses working in clinical roles were recruited. Among 501 eligible nurses, we selected the first 310 nurses who responded to the online questionnaire. The response rate was 61.9%. The sample consisted of 310 nurses from 29 hospitalization care units at 3 main hospitals in an autonomous region of Spain, Navarre. This sample size meets the recommendations for this type of analysis [18]. Participants were included if they had been working as nurses for at least one year. There were no restrictions for participation related to level of education.

#### 2.2.2. Data Collection

Data collection was conducted using SurveyMonkey, an online survey management platform where gathered data are transmitted over a Hyper Text Transfer Protocol Secure connection, and user logins are protected via Transport Layer Security (TLS). Data at rest are encrypted using industry standard encryption algorithms and strength. A time period between 10 and 15 min was estimated to complete the questionnaire. An online survey was created that included the 26 items of the Scope of Nursing Practice questionnaire and 3 sociodemographic questions related to age, years of nursing experience, and educational level. The link that gave access to the online questionnaire was successively distributed by e-mail to the nurses of the 29 hospitalisation units and until the responses of more than 300 participants had been obtained.

#### 2.2.3. Data Analysis

Univariate and multivariate normal distributions were evaluated for items. For univariate analysis, the Kolmogorov-Smirnov test and Mardia test were used for multivariate normal distribution. To describe the performance of each item, the estimated mean, standard deviation, median, and proportion for each level of response were calculated.

#### 2.2.4. Validity

For construct validity testing, two confirmatory factor analyses (CFA) were conducted to assess the goodness-of-fit to the Spanish version of the questionnaire using the factorial structure of the original six-factor structure [5] and a single factorial structure concerning validation in Arabic [13]. The fit of these two models was low, showing the following indices: CFI = 0.94, GFI = 0.80, and RMSEA = 0.089 for the original structure, and CFI = 0.92, GFI = 0.75, and RMSEA = 0.11 for the Arabic model fit.

Therefore, an exploratory factor analysis (EFA) was performed, to determine whether factor structure emerged for the Spanish ASCOP scale. The database was divided into two subsample groups using a random procedure and the analysis was performed on the data from Sample 1 (*n* = 155) assuming no a priori factor structure and including all 26 items. The sample was divided using a random procedure and both groups were compared to verify their homogeneity and that there were no significant differences between them. According to the ordinal nature of the data, the analyses were based on the polychoric correlation matrix, and principal factor axis factoring and promax rotation were used in an EFA to retain items by each factor. An item was chosen to load onto a specific factor if it achieved a simple structure, which was defined as the highest loading eigenvalue exceeding an absolute value of 0.40.

Then, a confirmatory factor analysis (CFA) was performed on the data from Sample 2 (*n* = 155) specifying the factor structure that had emerged in the EFA [18,19]. Several indices were used to test the goodness-of-fit: ratio, comparative fit index (CFI), the incremental fit index (IFI), the goodness-of-fit index (GFI), the adjusted goodness-of-fit index (AGFI), the Bentler relative noncentrality index (RNI), the Bentler–Bonett non-normed fit index (NFI), root mean square error of approximation (RMSEA), and the standardized root mean square residual (SRMR). Diagonally weighted least squares (DWLS) estimation was used, and indicators were modelled as ordered categorical variables [20].

Finally, the raw coefficient alpha was calculated from the total sample for the total score and subscale scores. Analyses were performed using IBM SPSS Statistics v. 25 software and R program (2021) for mac OS. The MVN package was used to estimate univariate and multivariate normality distribution. The psych and lavaan packages were used to conduct the EFA and CFA analyses, respectively.

#### 2.2.5. Ethical Issues/Statement

For the first phase of this study, permission to use and translate the instrument was obtained from Dr. D’Amour through e-mail correspondence. For the second part of this study, authorisation was obtained from the Committee of Ethics, Animal Experimentation, and Biosafety at the Public University of Navarre (PI:005/19). Participants gave free and informed consent to participate. The survey included a box on the first page of the questionnaire indicating whether the nurses agreed or not to participate in the investigation. Additionally, brief information on the study was incorporated. All data were anonymously analysed.

## 3. Results

The translation, back translation, and pilot test were conducted without major difficulties. The resulting version of the instrument is equivalent to the instrument in English from a linguistic point of view (see the Spanish questionnaire in Appendix A). 

The Kolmogorov-Smirnov test showed that no item met the assumption of a univariate normal distribution (*p*’s < 0.001, see Table 1). Additionally, the items-scale did not show multivariate normal distribution (Mardia Skewness = 0.47, *p* < 0.001; Mardia kurtosis = −0.365 *p* < 0.001). The mean, standard deviation, median, skewness, and kurtosis, as well as the normality parameters and the proportion of each response level, are displayed in Table 1.

### 3.1. Exploratory Factor Analysis

**Figure 1:**
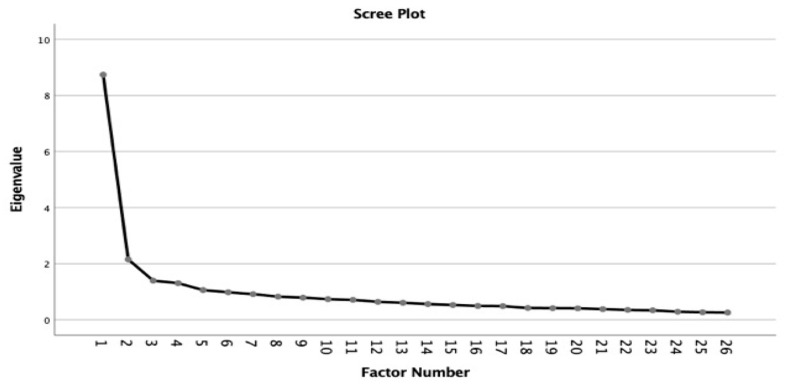
EFA Scree-plot.

To determine whether factor structure provided a better fit for the ASCOP Spanish version of the ASCOP, an EFA was conducted. The scree plot (see Figure 1) revealed a pronounced inflection point at the second-highest eigenvalue, with the first ten factors accounting for 42% of the cumulative variance, suggesting the adequacy of a two-factor model. Items 5, 15, 16, 17, 18, 21, 22, 24, 25, and 26 were loaded on the first factor with a simple structure and were labelled: “Patient and family centred care”. Another ten items (3, 4, 6, 7, 8, 9, 11, 13, 14, and 20) were loaded onto the second factor and were labelled as: “Quality of care and patient safety”. The remaining items (1, 2, 10, 12, 19, and 23) did not load with a simple structure onto any of the first two factors or were not part of a factor with enough items to compromise an independent subscale and were removed from the scale, yielding a final scale with twenty items and two subscales.

### 3.2. Confirmatory Factor Analysis

**Figure 2:**
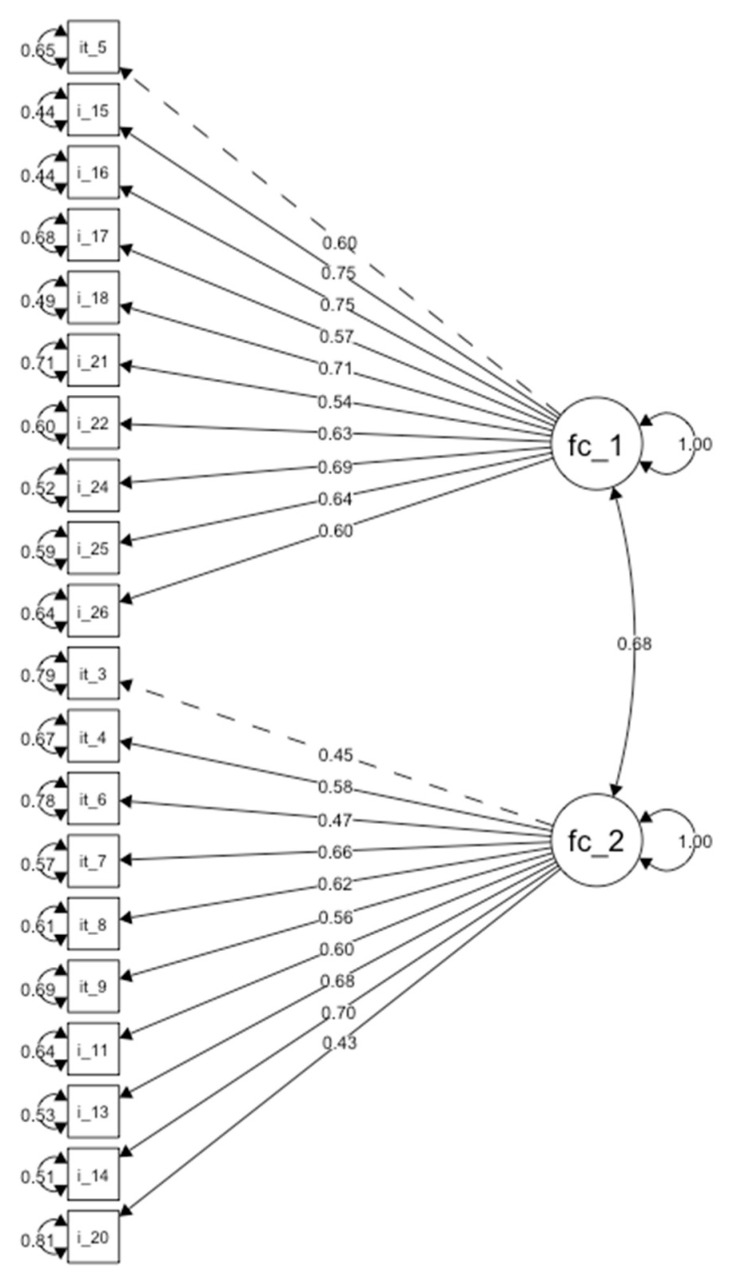
Confirmatory Factor Analysis path diagram.

A two-factor model fit emerged in the EFA and was examined using CFA in the other subsample of 155 participants. Diagonally weighted least squares estimation was employed, and indicators were modelled as ordered categorical variables [20]. The goodness-of-fit tests provided initial evidence that the two-factor solution was an adequate fit with the item scores, because the ratio of the x2/df was 1.6 (critical ratio cut-off of 2.0), CFI = 0.99, GFI = 0.96, AGFI = 0.957, and RNI = 0.996, which were all above 0.95 [21], the traditional cut-off establishing adequate fit. Other evidence that generally indicated that the two-factor solution was a good fit with the data was an RMSEA of 0.022 [90% CI = 0.000–0.044], and an SRMR = 0.077 (an RMSEA and SRMR < 0.06 to 0.08 indicate an adequate fit) [21]. Item loadings on their latent constructs were statistically significant (*p*’s < 0.001), suggesting that all items were optimally related to their respective latent construct. The values of the estimated parameters between latent constructs and items are presented in Table 2 and Table 3 and Figure 2.

A Cronbach’s α of 0.895 was obtained, demonstrating good internal consistency for and reliability of the Spanish version of the instrument, and this was the score for each of the subscales: a Cronbach’s α of 0.875 for the first factor and 0.825 for the second. Moreover, the item-total score adjusted correlation coefficients varied between 0.46 and 0.68.

Due to the lack of normality of the variables, the Spearman-Brown coefficient was also analyzed, showing r = 0.84 and demonstrating a good correlation and reliability.

## 4. Discussion

Defining nurses’ scope of practice in a specific health care context provides useful information to enhance the potential of the nursing workforce. To determine the scope of nursing practice, reliable and valid instruments are needed. This study was developed to provide a culturally-adapted and psychometrically sound instrument to establish the scope of nursing practice in the Spanish context. A new Spanish (spoken in Spain) version of the scale “Scope of Nursing Practice” with a good degree of validity and reliability is presented.

Among the different tools that exist to gauge the scope of nursing practice, the ASCOP instrument was selected, as it is the most widely used, with translations in French, English, and Arabic [5,13,14], and because it is for general nurses and not for a specific area. The process of translating and back-translating the ASCOP into Spanish was performed based on existing recommendations [16,17] to ensure content equivalence between the original and translated versions. The translation was performed in Spanish (spoken in Spain). Hence, further adaptation in terms of linguistic and cultural aspects might be necessary for the use of the Spanish version of the ASCOP in other Spanish-speaking countries in Central America and South America. A twenty-item Spanish version of the ASCOP, translated and modified from the original twenty-six-item English version of the ASCOP [5], was found to be valid and reliable, with two internally consistent factors.

Regarding the instrument’s internal consistency, the results showed a good level (raw coefficient alpha: 0.895), far exceeding the level of 0.70 suggested by Nunnally [22] and matching the original version (raw coefficient alpha: 0.89) [5]. The homogeneity of the statements was evaluated by means of the inter-item correlation coefficients and the corrected item-total score correlation coefficients, which revealed values above 0.30, the minimum level required for this type of correlation to be considered satisfactory according to Ebel and Frisbie’s criteria [23].

The results of the CFA indicated a poor fit for both the original six-factor model [5] and the single dimension identified in the Arabic translation [13]. Possible reasons for the poor fit with the original structure may include cultural or contextual differences already mentioned in relation to nurse competencies or activity organisation.

The two-factor structure was identified by an EFA with half of the sample, which was confirmed with a CFA including the other half. This procedure was proposed following the current recommendations to carry out a sequential use of EFA and CFA [18] based on previous research [19]. Thus, the sample is randomly divided into two subsamples and the EFA is performed with one half, and the resulting factorial structure is confirmed in the other half with a CFA. By such means, the factorial structure identified has greater robustness at the methodological level.

Furthermore, the two-factor structure could be considered adequate, as it explains 42% of the items’ cumulative variance in comparison with the original six-factor model, which explained 32.5%. There were no available data on the percentage of variance explained by the structure of the Arabic version of the ASCOP. The first factor comprised direct care interventions. This factor was labelled as “Patient and family centred care” and included items (5, 15, 16, 17, 18, 21, 22, 24, 25, and 26) that described activities related to patient and family education and assessment, coordination and continuity of care, and evidence-based practice. The second factor included indirect care interventions that ensure quality of care and patient safety, which is why it was labelled as “Quality of care and patient safety”. This factor contains items (3, 4, 6, 7, 8, 9, 11, 13, 14, and 20) that represented activities concerning teamwork, mentorship and training within the nursing team, nursing care protocol development/implementation, and knowledge updating.

For many years, different studies have determined the existence of direct care activities and indirect activities within the work carried out by hospital nurses [24,25]. This typology is also used as a differentiating element of nursing interventions, as defined in the internationally known Nursing Interventions Classification (NIC) [26].

The scale factor clustering direct care activities is related to three of the five functions of the nursing role identified by Dallaire and Dallaire [27], caring, coordinating, and educating, while the factor that groups indirect care activities was related to the other two functions (supervising and collaborating). This factor associated with indirect care activities also refers to functions not so closely related to the clinical dimension of nursing practice, such as the use of evidence and leadership, which are described in the professional role model of O’Rourke [28].

In addition, it should be emphasised that the nature of nurses’ activities according to their classification as direct or indirect is one of the variables to consider from an ethical point of view in regard to resource allocation and rationing [29]. Thus, when rationalising work, nurses attempt to balance indirect with direct patient care [30].

Based on the EFA performed, six items from the original scale were excluded from the Spanish version as they did not fit into the two identified factors (i.e., 1, 2, 10, 12, 19, and 23). In any of these items, with regard to their classification and the two factors identified, it would be possible to include them as both direct and indirect care activities.

One of the most important points in this research is to consider the special characteristics of nurses in Spain. It is well known that each country has its own particularities both in terms of training and practice. Nursing education in Spain has been brought up to the standards of the European Higher Education Area following the implementation of the “Bologna Plan”. University degree programmes in nursing in Spain are at a higher level than in other countries in Europe and the world. This is mainly because nursing in Spain is considered a profession with a high professional level. This is likely due to the high undergraduate qualification with the option of access to master’s and postgraduate studies, which is not possible in all countries.

It should be noted that the contextual differences between countries hinder the unification of criteria in the definition of the nursing role [31]. For example, according to this article, the competencies between a nurse in France and a nurse in Turkey can be completely different. As such, the differences we identify in the Spanish context will be critical when defining the activities that nurses perform and establishing the scope of nursing practice.

All the units included in the study were selected for their similar characteristics, excluding the maternity and paediatric hospitalization units. However, the consideration of units with similar characteristics, but which could be classified as medical or surgical, could be interesting to observe if there are differences in their respective nurses’ scope of practice. Future analysis should consider incorporating this.

The chief limitation of this study is not having obtained an outcome in accordance with the original scale in terms of structure. It is not easy to build a competency framework that considers the particularities of nurses in all countries. Although we did not derive an identical result, we were able to obtain an instrument that collects information on the activities that nurses carry out, and which defines the scope of nursing practice.

Additional studies should be conducted in different caregiving populations in Spain and Europe to determine whether the two-factor structure still holds. We suggest being conservative with the results due to the regional nature of the survey, and also because the questionnaire was completed online, where people are more likely to respond.

On the other hand, this article paves the way for future research. It would be interesting to analyse and quantify the activities that nurses carry out that are “not specific” to their scope of practice. In that way, causal relationships could be established between the full implementation of the scope of practice and whether their correlation to the performance of other tasks does not add professional value. There has been an increase in the literature demonstrating the importance of the need for nurses to work within their full scope of practice [32]. Hence, future studies should relate the level of implementation of the scope of practice to nursing ratios in different countries.

## 5. Conclusions

This study contributes new knowledge by presenting the first Spanish (spoken in Spain) version of the ASCOP questionnaire. The Spanish version of the questionnaire showed a good level of internal consistency, and a two-factor structure was confirmed. One of the factors comprised direct care interventions (patient and family centred care) and the other one included indirect care interventions (quality of care and patient safety).

The Spanish version of the ASCOP is a valid instrument by which to measure the scope of nursing practice and to assess the nature of the activities carried out by nurses. This questionnaire determines the level of complexity of these activities, thus providing a differential perspective in relation to other instruments.

Having a tool adapted to the Spanish context to identify to what level nurses are fulfilling their scope of practice is essential to guide the correct decisions in nursing human resource policies.

## Figures and Tables

**Table 1:** Descriptive, Statistic of Normality, and Proportion for each level of response by item.

	Descriptive Information					Statistic for Normality			Proportion for Each Level of Response					
	Mean	Std. Dev	Median	Skew	Kurtosis	Lilliefors (KS)	*p* Value	Normality	1	2	3	4	5	6
item_1	4.29	1.134	4	−0.073	−0.832	0.174	<0.001	NO	0.3	0.3	3.5	24.2	28.1	26.8
item_2	4.33	1.275	4	−0.203	−1.003	0.165	<0.001	NO	0.3	0.3	7.7	21	24.5	22.6
item_3	3.3	1.187	3	0.408	−0.236	0.2	<0.001	NO	3.5	3.5	23.2	33.2	25.2	9.4
item_4	3.9	1.006	4	0.209	−0.056	0.23	<0.001	NO	0.3	0.3	6.5	27.1	43.2	15.2
item_5	4.33	1.074	4	−0.11	−0.413	0.213	<0.001	NO	0.3	0.3	3.9	16.1	39	23.9
item_6	3.9	1.182	4	−0.124	−0.294	0.169	<0.001	NO	2.6	2.6	8.1	25.8	33.5	20.3
item_7	3.96	1.175	4	0.106	−0.37	0.202	<0.001	NO	1.6	1.6	6.5	28.4	35.2	14.8
item_8	3.55	1.383	4	0.044	−0.718	0.153	<0.001	NO	6.8	6.8	18.1	22.6	28.7	13.5
item_9	3.91	1.23	4	−0.205	−0.266	0.181	<0.001	NO	3.5	3.5	8.4	22.9	34.8	19.4
item_10	4.21	1.157	4	−0.126	−0.648	0.175	<0.001	NO	0.3	0.3	7.1	19.4	33.5	23.9
item_11	3.28	1.35	3	0.346	−0.579	0.174	<0.001	NO	7.1	7.1	24.8	27.1	22.6	10.3
item_12	4.02	1.415	4	−0.378	−0.631	0.171	<0.001	NO	5.5	5.5	11	15.8	28.4	22.3
item_13	3.34	1.438	3	0.271	−0.704	0.156	<0.001	NO	9	9	24.2	20.3	28.4	6.8
item_14	3.38	1.392	3	0.157	−0.731	0.152	<0.001	NO	8.7	8.7	20.3	25.5	23.9	13.2
item_15	3.42	1.596	3	0.077	−1.103	0.138	<0.001	NO	14.2	14.2	18.4	20.3	18.4	16.1
item_16	4.54	1.311	5	−0.644	−0.22	0.174	<0.001	NO	2.6	2.6	4.5	13.5	25.8	22.9
item_17	5.12	0.962	5	−0.784	−0.09	0.279	<0.001	NO	0	0	1.3	2.9	24.5	25.5
item_18	4.34	1.153	4	−0.084	−0.678	0.211	<0.001	NO	0.3	0.3	4.8	17.4	37.1	19
item_19	4.07	1.168	4	0.064	−0.528	0.201	<0.001	NO	1	1	6.1	25.5	35.2	17.1
item_20	3.52	1.331	3	0.112	−0.494	0.165	<0.001	NO	6.8	6.8	14.5	30	27.1	11.9
item_21	5.01	1.149	5	−1.056	0.47	0.264	<0.001	NO	0.3	0.3	4.5	4.8	20.6	23.9
item_22	4.42	1.214	4	−0.375	−0.551	0.173	<0.001	NO	1	1	5.2	16.8	28.1	25.8
item_23	3.31	1.313	3	0.244	−0.632	0.155	<0.001	NO	6.5	6.5	24.5	25.2	25.5	11.9
item_24	4.35	1.382	4	−0.445	−0.638	0.162	<0.001	NO	2.6	2.6	9	13.5	28.7	18.4
item_25	5	1.049	5	−0.745	−0.206	0.259	<0.001	NO	0	0	2.3	5.2	25.8	23.9
item_26	4.03	1.351	4	−0.17	−0.791	0.142	<0.001	NO	2.6	2.6	11.9	20.3	27.7	19.7

Note: KS = Kolmogorov-Smirnov.

**Table 2:** EFA and CFA Factor Loadings.

	EFA			CFA			
	Factor Loading			95% CI			
Kiss Item	1	2	λ	LL	UL	SE	*p* Value
item_1R	0.262	0.195					
item_2R	0.137	0.184					
item_3	−0.045	0.539	1.116	0.855	1.376	0.133	<0.001
item_4	0.049	0.419	0.661	0.475	0.847	0.095	<0.001
item_5	0.495	0.158	0.751	0.548	0.955	0.104	<0.001
item_6	−0.189	0.716	1.065	0.795	1.334	0.138	<0.001
item_7	−0.060	0.575	0.745	0.519	0.970	0.115	<0.001
item_8	−0.126	0.669	1.165	0.828	1.501	0.172	<0.001
item_9	−0.014	0.581	1.005	0.736	1.275	0.138	<0.001
item_10R	0.377	0.355					<0.001
item_11	0.044	0.460	1.153	0.824	1.482	0.168	<0.001
item_12R	0.331	0.349					
item_13	0.168	0.512	1.057	0.770	1.345	0.146	<0.001
item_14	−0.035	0.626	0.968	0.637	1.298	0.169	<0.001
item_15	0.531	0.103	1.162	0.788	1.537	0.191	<0.001
item_16	0.673	0.041	0.846	0.587	1.105	0.132	<0.001
item_17	0.810	−0.129	0.629	0.484	0.774	0.074	<0.001
item_18	0.521	0.279	0.694	0.509	0.879	0.094	<0.001
item_19R	0.325	0.362					
item_20	−0.143	0.651	1.322	0.971	1.672	0.179	<0.001
item_21	0.667	−0.263	0.990	0.772	1.208	0.111	<0.001
item_22	0.764	0.027	0.824	0.622	1.026	0.103	<0.001
item_23R	0.280	0.209					
item_24	0.630	−0.051	1.010	0.701	1.318	0.157	<0.001
item_25	0.706	−0.112	0.648	0.472	0.824	0.090	<0.001
item_26	0.444	0.320	1.227	0.939	1.516	0.147	<0.001

Note: EFA = Exploratory Factor Analyses; CFA = Confirmatory Factor Analyses; SE = Standard Error; CI = confidence interval; LL = lower limit; UL= upper limit.

**Table 3:** CFA Adjustment Indices.3.1.1. Subsubsection.

Adjustment Indices		Good Fit	6 Factors	1 Factor	2 Factors
Reason For Fit	χ2/gL	<2			1.6
Root Mean Square Error of Approximation	RMSEA	<0.08	0.09	0.11	0.02
Standarized Root Mean Square Residual	SRMR	<0.08	0.11	0.12	0.07
Goodness-of-Fit Index	GFI	>0.90	0.80	0.75	0.96
Comparative Fit Index	CFI	>0.90	0.94	0.92	0.99

## Data Availability

Data sharing is not applicable to this article.

## Appendix A

“ASCOP questionnaire” in Spanish (Cuestionario del Alcance de Práctica de Enfermería).

Instrucciones: Indique para cada enunciado la respuesta que mejor corresponda a su práctica diaria en su puesto de trabajo. Seleccione la frecuencia con la que realiza estas actividades: . 1. Nunca . 2. Muy raramente . 3. A veces . 4. Frecuentemente . 5. Casi a diario . 6. Siempre						
**Ítems**	**1**	**2**	**3**	**4**	**5**	**6**
Participo en el desarrollo de la práctica enfermera. (ej. Revisión de protocolos de cuidados, proyectos diversos…)						
Mantengo al día mis conocimientos. (lectura de artículos científicos, etc.)						
Utilizo estrategias didácticas que se adaptan a cada paciente y a sus familiares según el nivel de autonomía del paciente.						
Ejerzo de mentora o formadora del personal de reciente incorporación.						
Notifico las situaciones clínicas en las que considero que existen deficiencias respecto a la calidad y a la seguridad de los cuidados.						
Participo en reuniones y actividades del equipo interdisciplinar						
Participo en la orientación y la formación de estudiantes de enfermería o del personal de reciente incorporación						
Participo en la creación, la aplicación y la actualización de distintos planes y protocolos de atención a los pacientes (por ejemplo: Planes de cuidados estandarizados, informes de enfermería al alta, etc.)						
Cuando se realiza la modificación de una práctica, comparto con el equipo de enfermería la evidencia derivada de los estudios de investigación.						
Participo en el desarrollo y realización de actividades formativas del equipo de cuidados en función de mi capacidad.						
Para asegurar la continuidad de los cuidados, coordino las intervenciones del equipo interprofesional en el hospital (médicos, fisioterapeutas, etc.)						
Comunico a los miembros del equipo toda la información que podría afectar a la coordinación de la atención.						
Verifico que el paciente y sus familiares han comprendido las indicaciones que se les han facilitado.						
Mejoro mi práctica basándome en nuevos conocimientos derivados de las “buenas prácticas” e investigaciones en enfermería y otras ciencias de la salud.						
Participo en la identificación de las necesidades de formación continua de mi unidad.						
Actualizo con regularidad, por escrito, la información sobre el estado del paciente y los cuidados que se le proporcionan (el plan de cuidados, las anotaciones de las enfermeras, etc.).						
Compruebo la calidad de la información facilitada al paciente en la unidad.						
Transmito toda la información relevante a los profesionales sanitarios de otras instituciones para asegurarme de la continuidad de los cuidados.						
Evalúo el estado físico y mental del paciente teniendo en cuenta los aspectos biopsicosociales.						
Participo en la actualización de las prácticas para mejorar la calidad y la seguridad de los cuidados.						
